# Symposium: Risk, innovation and ignorance production in the field of reproductive biomedicine

**DOI:** 10.1016/j.rbms.2021.11.003

**Published:** 2021-11-24

**Authors:** Susan E. Bell

**Affiliations:** Department of Sociology, Drexel University, Philadelphia, PA, USA

**Keywords:** Undone science, Ignorance studies, Ignorance production, Non-knowledge, Feminist ignorance epistemologies

## Abstract

This commentary evaluates the contributions to this special issue through a feminist lens. It reviews the field of ignorance studies and two distinguishable areas in the field: undone science and ignorance/non-knowledge. It points to ways in which the articles in this special issue engage with the social production of ignorance. It explores feminist roots of ignorance studies, including feminist ignorance epistemologies, identifies strengths and gaps in ignorance studies, and suggests possible lines of future work.

## A feminist introduction

It is a pleasure to comment on the contributions to this special issue, 'Risk, Innovation and Ignorance Production', and to consider them through a feminist lens. In the 1970s and 1980s feminist scholars and activists began to explore the issues of what is now called 'undone science' and 'ignorance studies' (e.g., Martin, 1987) and proposed the concept of 'situated knowledge' (Haraway, 1988), but it was Nancy Tuana, 2004, Tuana, 2006 who named and set forth a taxonomy of ignorance as part of a proliferating field of studies with which the current papers engage. In this commentary, I review the field of ignorance studies, weave in feminist strands, identify strengths and gaps, and suggest possible lines of future work. Throughout, I point to ways in which the articles in this collection highlight and engage with the social production of ignorance.

There are two big overlapping but distinguishable areas in the field, undone science, which emphasizes the role of social movements and politics, and ignorance/non-knowledge, which emphasizes how modern and reflexive modernity have brought attention to and complicated the production of ignorance. Both agree that ignorance is produced alongside of knowledge and that ignorance is a routine part of knowledge-production. Both focus on a wide range of cases (e.g., global financial crisis, climate change, GMOs, ecological design, chlorine science, community-based air monitoring, and environmental causes of breast cancer).

The field of ignorance studies has emphasized research emanating from and analyzing topics situated in North America and Europe. Similarly, with the exception of Maffi’s study of medical abortion in Tunisia, the papers in this collection examine the dynamics of undone science and ignorance in the UK and Europe. Overall, inquiry into undone science and ignorance has underemphasized gender and reproductive technologies and practices. This body of scholarship also ignores early feminist work that tackled questions of undone science and sexism (see, Martin, 1987, Boston Women’s Health Book Collective, 1973, Tuana, 2004). The papers in this collection help to redress the imbalance by unpacking undone science and the construction of ignorance in the development of pregnancy tests by injection beginning in the 1940s (Nemec and Olszynko-Gryn), technologies of pregnancy and childbirth (Topçu, Mirouse, Fillion and Torny), new reproductive technologies (Herbrand), medical abortion (Maffi), and the diagnosis and treatment of endometriosis (Hudson).

## Feminist roots of ignorance studies

Feminists have a long history of highlighting the ignorance that is produced by sexism. In 'The Egg and the Sperm', Martin (1991: 486) 'shines a bright light on the gender stereotypes hidden within the scientific language of biology' that produced ignorance about human fertilization. And just as ignorance is useful or profitable for the powerful, it can also be emancipatory for the less powerful. In the 1970s, 'epistemologies of ignorance' were key strategies of the women’s health movement, e.g., the Boston Women’s Health Book Collective’s *Our Bodies, Ourselves* (BWHBC, 1973)*.* These epistemologies of ignorance included uncovering the ways women’s bodies had been ignored, examining knowledge that had been withheld from women, reclaiming knowledges that had been denied, suppressed, or erased, and developing new knowledges (Tuana, 2006:2). In a study of the premarketing review of the anti-miscarriage drug DES (diethylstilbestrol) before its release for sale in the USA in 1941, I demonstrated how warnings of its dangers to women’s bodies were suppressed and set aside by American scientists and physicians, federal regulators, and pharmaceutical manufacturers (Bell, 1986). In subsequent research, I focused on the production of ignorance about transplacental effects of DES that caused reproductive tract cancer and infertility and how, beginning in the 1970s, an embodied health movement of DES daughters and mothers successfully brought their experiences into focus and helped to direct a new research agenda by the 1990s (Bell, 2009). Other examples of epistemologies of ignorance in feminist research on health include the menstrual cycle (Martin, 1987), orgasm (Tuana, 2004), and female sexual anatomy (Moore and Clarke, 1995).

Early feminist ignorance epistemologies in the USA flowed between academic and activist settings. Black Feminist Health Science Scholars have renewed the practice in the 21st century, moving 'beyond the pages of journals to practical application in the world' (Bailey and Peoples, 2017:17). In 1994 feminist ignorance epistemologies were repositioned by an intersectional reproductive justice framework that integrated race, gender, and class oppressions (Roberts, 2015) and, increasingly, focused on the role Black women’s bodies have played in the development of biomedical science and the co-constitutive nature of medical science and popular perception (Bailey and Peoples, 2017). Despite tackling questions of undone science and the production of ignorance, these two areas of scholarship have emerged alongside feminist inquiry but not in dialogue with it.

## Undone science

The study of undone science emerged in the 1970s and 1980s, when STS scholars looked at institutional and political factors shaping scientific fields and practices (Frickel et al., 2010). Multiple and competing groups – academic scientists, government funders, industry, and civil society organizations – struggle over the construction and implementation of research agendas. Undone science consists of 'areas of research that are left unfunded, incomplete, or generally ignored but that *social movements* or *civil society organizations* [sic] often identify as worthy of more research' (Frickel et al., 2010: 444). That is, it refers to 'the systematic absence of research identified by counter publics when they seek to document potential risks and uncertainties of technologies and industrial processes, and they find that the desired research has not been done or has been significantly underfunded' (Hess, 2016: 2). Often the systematic absence is matched by a much higher quantity of industry-funded research supporting the safety and efficacy of technologies and products.

Ignorance is tied to places, 'domains of imperceptibility or knowledge gaps … that can be mapped across space' (Frickel and Kinchy, 2015: 180). Feminist scholars and women’s health movements, for example, showed gendered ignorance about women’s bodies produced in laboratories, hospitals, and clinics (e.g., Bell, 1994, Bell, 1995). In her study of endometriosis, Hudson contrasts accounts by women of their embodied experiences with practices of ignorance in white androcentric medicine. Gendered oppression and the exclusion of women from agenda setting and places of knowledge has produced ignorance about women’s pain and suffering. Hudson points to new global feminist movements of menstrual health, such as period politics, as hopeful signs against the invisibilizing and whitening of endometriosis.

A key contribution of undone science is that it brings attention to the distribution of power and access to resources that enables research deemed worthy and important and blocks science that is deemed unimportant, too dangerous, or socially harmful to the politics of doing science. In other words, the concept of 'undone science' is a tool for making visible and understanding the uneven distribution of power and resources in science and at the same time for understanding how knowledge and ignorance are socially shaped, constructed, and contested.

Several papers in this special issue focus on power, resources, and the social construction of ignorance. In their study of the post-World War II debate in West Germany about reproductive risk and disability Nemec and Olszynko-Gryn show the production of ignorance about Duogynon, a hormonal test for pregnancy, was haunted by thalidomide and National Socialism in the struggle among multiple and competing groups and individuals from the pharmaceutical industry, patient groups in Germany and the UK, medical experts, and media. As they write, knowledge and ignorance – about pharmaceuticals, adverse effects, and iatrogenic birth defects – were structurally produced and maintained, with the help of influential experts and powerful networks. In their study of a different synthetic hormone, Fillion and Torny highlight the dynamics of ignorance in France about the transgenerational effects of prenatal exposure to DES (diethystilbestrol) that began after publication of a case-control study by American physicians in 1971. Fillion and Torny identify strategic ignorance among French doctors about the effects of DES on the children and grandchildren of women who were prescribed it during pregnancy, produced by doctors’ disqualification of local clinical knowledge, marginalization of pioneering French women obstetricians, ignored knowledge from the USA and Netherlands, and the disqualification of patients’ and exposed persons’ embodied knowledge. Social movements of families and their clinical allies developed a variety of evidence-based activism and their judicial strategies secured social recognition of DES effects.

Two other papers also explore the dynamics of undone science among groups with unequal access to power and resources. Mirouse shows the production of ignorance about episiotomy in professional journals by/for obstetricians and midwives in France during the late 20th century. The contest between elite and non-elite professionals led to the routinization of episiotomy in France, despite recommendations against its routine use by the World Health Organization. Herbrand identifies a group of powerful actors – scientists, clinicians, patient support groups, and the Wellcome Trust – that successfully deployed a wide array of strategic actions against a weak and disorganized counter public to produce ignorance about mitochondrial donation. Approved by the British Parliament in 2015, mitochondrial donation involves transferring the nucleus of an affected embryo into a healthy donor embryo (from which the nucleus has been removed). By focusing attention on some known unknowns, these actors obscured other known unknowns. They produced ignorance about what Herbrand calls 'ignored knowns', limits to mitochondrial donation that were known but made invisible.

Focusing on gender and reproduction, each of these papers draws out the social factors in the production of ignorance and the dynamics of power among groups and individuals. In three cases they resulted in the production and continued use of reproductive technologies that were harmful to women and/or children.

## Ignorance/non-knowing

Ignorance is a kind of cover term that points to the borders and limits of knowing, or non-knowledge, and it includes the intentional or unintentional 'bracketing out of unknowns' (Gross, 2007: 249). An important contribution of this approach is its characterization of knowledge in contemporary society as a contingent and fragile achievement and non-knowing as an inescapable condition. (Beck and Wehling, 2011: 41). In their Introduction to the *Routledge International Handbook of Ignorance Studies* Gross and McGoey (2015: 1) write that since 2000 'the terrain of ignorance studies has developed into a dynamic field that has forged links across many disciplines … to explore the social life and political issues involved in the distribution and strategic uses of not knowing.'

Gross and McGoey, unlike many others in this field, draw inspiration from postcolonial and feminist theorists’ work. 'White' or 'willful ignorance' is useful non-knowledge for dominant groups for denying, justifying, or simply ignoring 'the reality of past and present atrocities against the less powerful' (Gross and McGoey, 2015: 4-5). For example, unknowing is a condition of scientific knowledge in large-scale medical research sites that link African laboratories and hospitals to North American or European scientific agencies (Geissler, 2013). The experiences of material differences across the scientific trial community, both for study participants and research workers, justify the research and are simultaneously unknown. 'Unknowing serves to make scientific collaboration feasible [because it links] bodies, lives, institutions, funding, and technologies across wide differentials of resources, expertise, and power' (Geissler, 2013: 17).

In her contribution to this special issue, Maffi explores the strategic uses of not knowing in the production of ignorance about medical abortion in Tunisia by actors in medicine, law, and religion. Against the background of post-colonial Tunisia’s decriminalization of abortion in 1973, she focuses on 2001, when medical abortion was approved to the mid-2010 after the revolution of 2011 when very conservative religious repertoires about women’s status, rights, and sexual conduct emerged. Ignorance was accomplished by mainly male gynecologists, jurists, and politicians, through a lack of training, work conditions, the moral or religious stance of providers, conservative religious repertoires, and multiple Islamic positions about abortion that became significant after 2011.

Topçu picks up the feminist theme of erasure, or what she calls 'technology-driven ignorance', about pain and pain relief in post-1968 France that resulted in the routinization of epidural analgesia (EA) during childbirth. Medicine was the dominant discourse, and obstetricians and anesthesiologists were the primary actors. Gradually and actively, common knowledge about giving birth without medication disappeared. When feminists weighed in, they generally supported the introduction of pharmaceutical pain relief and criticized natural childbirth movements (birth without pain, birth without violence) as patriarchal, and thus feminists contributed to the medicalization of childbirth, including use of EA; more recently some feminists have denounced EA as a case of obstetric violence. Topçu complicates feminist epistemologies of ignorance by showing there are not always clearly demarcated 'sides' and illustrating the fragility and contingency of knowledge.

## Feminist reflections

In her recent reflections about feminist women’s health in the 21st century, Adele Clarke (2021: 36) laments that 'sexism in medicine continues to be endemic and horrifically consequential for women.' Her lament resonates with the cases reported in this special issue, ranging from the routinization of episiotomy in France to the difficulty of gaining access to medical abortion in Tunisia or pain relief from endometriosis in the UK, and the continued use of harmful pharmaceuticals before and during pregnancy. At the same time, these papers add depth and complexity to understanding the intricate entanglements of knowledge and ignorance production and the dangers of assuming it is always possible to identify 'sides'.

More broadly, the field of ignorance and undone science would profit from recognizing and continuing to flesh out feminist theorists’ work, as is the case here. In addition, it would benefit from more studies that, like Maffi’s, are inspired by a postcolonial framework and, from a global or transnational perspective, look at forms of unknowing that weave together local dynamics, national politics, global treaties, state institutions, donor foundations, UN agencies, and national and international non-governmental organizations (e.g., Geissler, 2013, Suh, 2021). Because all knowledge is situated, 'ignorance is an inevitable consequence' (Gross and McGoey, 2015: 4-5). There is still much to know about ignorance and undone science in reproductive medicine. In its production of knowledge about ignorance, this special issue opens up new and exciting pathways.

## Declaration

The author reports no financial or commercial conflicts of interest.
